# Management of severe hypercalcaemia secondary to primary hyperparathyroidism: The efficacy of saline hydration, furosemide, and zoledronic acid

**DOI:** 10.1002/edm2.380

**Published:** 2022-10-04

**Authors:** Ibtissem Oueslati, Asma Kardi, Meriem Yazidi, Sahar Abidi, Fatma Chaker, Seifeddine Mellassi, Melika Chihaoui

**Affiliations:** ^1^ Department of endocrinology, Faculty of Medicine, La Rabta University Hospital University of Tunis‐El Manar Tunis Tunisia

**Keywords:** bisphosphonates, emergency, furosemide, primary hyperparathyroidism, saline hydration, severe hypercalcaemia

## Abstract

**Introduction:**

Severe hypercalcaemia is a life‐threatening condition that should be managed urgently. The aim of this study was to assess the efficacy of saline hydration, furosemide, and zoledronic acid in the management of severe hypercalcaemia secondary to primary hyperparathyroidism (PHPT).

**Methods:**

We conducted a retrospective analysis of the management of 65 patients with severe hypercalcaemia (≥3 mmol/L) secondary to PHPT. The efficacy of each therapeutic agent was evaluated according to the variation in serum calcium level calculated as Δ calcium = initial calcium level – minimal calcium level reached after the administration of each agent.

**Results:**

The mean age of patients was 56.4 ± 13.8 years. At baseline, the mean total serum calcium level was 3.42 ± 0.40 mmol/L. After normal saline hydration, calcium level decreased from 3.25 ± 0.21 to 2.98 ± 0.2 mmol/L (*p* < 10^−3^) in 3.1 ± 1.7 days. Normalization of calcium level did not occur in any patient. Furosemide was prescribed in 35 patients. It resulted in a serum calcium increase of 0.09 ± 0.21 mmol/L. Calcium levels did not reach the normal range in any patient. Forty‐five patients received intravenous zoledronic acid. The mean maximal reduction in serum calcium level was 0.57 ± 0.27 mmol/L (from 3.25 ± 0.26 mmol/L to 2.68 ± 0.22 mmol/L, *p*‐value <10^−3^). Normalization of calcium levels occurred in 27 patients (60%).

**Conclusions:**

Our results show the absence of a significant additional effect of furosemide on calcium levels in patients with severe hypercalcaemia secondary to PHPT when compared with the effect of saline hydration alone. However, zoledronic acid was more potent. Thus, appropriate normal saline hydration with immediate intravenous bisphosphonates infusion should be considered in the management of severe hypercalcaemia in patients with PHPT.

## Graphical abstract

Appropriate normal saline hydration with immediate intravenous bisphosphonates infusion should be considered in the management of severe hypercalcemia in patients with primary hyperparathyroidism.

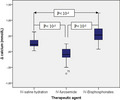

## 1 INTRODUCTION

Severe hypercalcaemia in the setting of primary hyperparathyroidism is called hyperparathyroid crisis. Its incidence ranges from 1.6% to 6%.^1^, ^2^ Presentations of hyperparathyroid crisis are heterogeneous including neurological alteration, dehydration, hypovolaemia, anorexia, vomiting, cardiac arrhythmia, impaired cardiac, and renal functions, and finally death if the condition is untreated.^3^ Thus, the parathyroid crisis should be managed urgently. Parathyroidectomy represents today its only curative treatment option. However, preoperative medical antihypercalcaemic therapy is warranted as a holding measure. It consists of intravenous saline hydration, a loop diuretic, intravenous administration of bisphosphonates, and calcimimetics. Data evaluating the efficacy of these treatments are very limited.

The aim of this study was to assess the efficacy of saline hydration, furosemide, and zoledronic acid in the management of severe hypercalcaemia secondary to primary hyperparathyroidism.

## 2 METHODS

This was a retrospective, single‐centre study including patients with severe hypercalcaemia secondary to primary hyperparathyroidism who were admitted between January 2012 and March 2021. Inclusion criteria were patients with primary hyperparathyroidism aged more than 18 years, with a corrected total serum calcium level ≥3.5 or ≥3 mmol/L with signs of poor tolerance (confusion, lethargy, coma, dehydration, electrocardiogram changes, acute renal failure, etc.), and who received a symptomatic treatment before surgery. Non‐inclusion criteria were chronic severe renal failure, heart failure, pregnancy, lactation, and other causes of hypercalcaemia.

The diagnosis of primary hyperparathyroidism was established in the presence of hypercalcaemia with elevated or inappropriately normal parathormone (PTH) concentration.

A total of 65 patients were enrolled in this study. Demographic, clinical (anthropometric parameters, hydration state, neurological signs, blood pressure, cardiac rate, and diuresis), and baseline biochemical (serum calcium, serum phosphate, albumin, urea, creatinine, PTH, and 25 OH‐vitamin D levels) data were collected. The albumin‐corrected calcaemia was calculated in patients with albuminaemia levels <40 g/L according to the following formula: total calcaemia (mmol/L) + 0.02 × (40 − albuminaemia [g/L]). Creatinine clearance was estimated using the MDRD (Modification of Diet in Renal Disease) equation. Renal failure was defined as a creatinine clearance of <60 ml/min and severe renal failure by a creatinine clearance of <30 ml/min.

Cervical ultrasound, technetium‐99m sestamibi scintigraphy, cervicothoracic computed tomography scan, renal ultrasound, and bone mineral densitometry results were recorded.

The dose and the duration of each therapeutic agent prescribed (saline hydration, furosemide, and zoledronic acid) were noted in each patient. The infusion rate of saline hydration was adjusted according to the patient's age and comorbidities, hydration status, and serum calcium levels. Furosemide was administrated after proper hydration by saline solution with adequate control and supplementation of electrolytes. Intravenous zoledronic acid was used in patients whose calcium levels remained ≥3 mmol/L, despite appropriate saline hydration alone or with furosemide administration.

The efficacy of each therapeutic agent was evaluated according to the variation in serum calcium level calculated as Δ calcium = calcium level before the administration of each therapy –minimal calcium level after. Normalization of serum calcium was defined by a level of <2.6 mmol/L.

### 2.1 Statistical analysis

Data were analysed using SPSS (Statistical Package for the Social Sciences) version 24. Categorical variables were presented as percentages and continuous variables as mean ± standard deviations. The comparisons between quantitative variables were made using Student's *t*‐test and in case of non‐validity by the non‐parametric Mann–Whitney test. The level of significance was set at 0.05.

## 3 RESULTS

Sixty‐five patients (58 women and 7 men) were enrolled in this study. Their mean age was 56.4 ± 13.8 years [extremes: 21–89]. Hypercalcaemia was incidentally discovered in 29% of cases, in the presence of renal, bone, digestive, and cardiac manifestations in 38%, 14%, 5%, and 2% of cases, respectively, and impaired general condition in 12% of patients.

The baseline characteristics of patients are shown in Table 1.

**TABLE 1:** Baseline characteristic of the study population.

	Patients data
Mean age (years)	56.4 ± 13.8
Sex‐ratio (women/men)	58/7
Smoking (%)	6
Hypertension (%)	15
Diabetes mellitus (%)	12
Obesity (%)	23
Symptoms and signs (%)	
Asthenia	32
Anorexia	8
Weight loss	18
Nausea‐vomiting	5
Abdominal pain	10
Constipation	8
Neuropsychiatric manifestations	8
Polyuria‐polydipsia	25
Dehydration	16
ECG changes	17
Renal colic	11
Bone pain	32
Serum biochemical tests (mean ± SD)	
Calcium (mmol/L)	3.42 ± 0.40
Albumin (g/L)	36,5 ± 5,3
Phosphate (mmol/L)	0.62 ± 0.17
Calcium/phosphate ratio	5.51 ± 2.35
PTH (ng/L)	870.7 ± 795.5
Urea (mmo/L)	5.7 ± 3.5
Creatinine (μmol/L)	78.7 ± 1.8
Calciuria (mmol/kg/24 h)	0.16 ± 0.08
Vitamin D deficiency (%)	96
PHPT complications (%)	
Nephrolithiasis	34
Nephrocalcinosis	0
Renal failure	23
Osteoporosis	73
Pancreatitis	5

Abbreviation: PHPT, primary hyperparathyroidism.

At admission to our department, the mean albumin‐corrected serum calcium level was 3.42 ± 0.40 mmol/L with extremes of 3 and 5.4 mmol/L. Twenty‐two patients had calcaemia ≥3.5 mmol/L and 43 patients had calcaemia ≥3 mmol/L with acute renal failure (*n* = 18), electrocardiogram changes (*n* = 17), neurological symptoms (*n* = 8).

**FIGURE 1:**
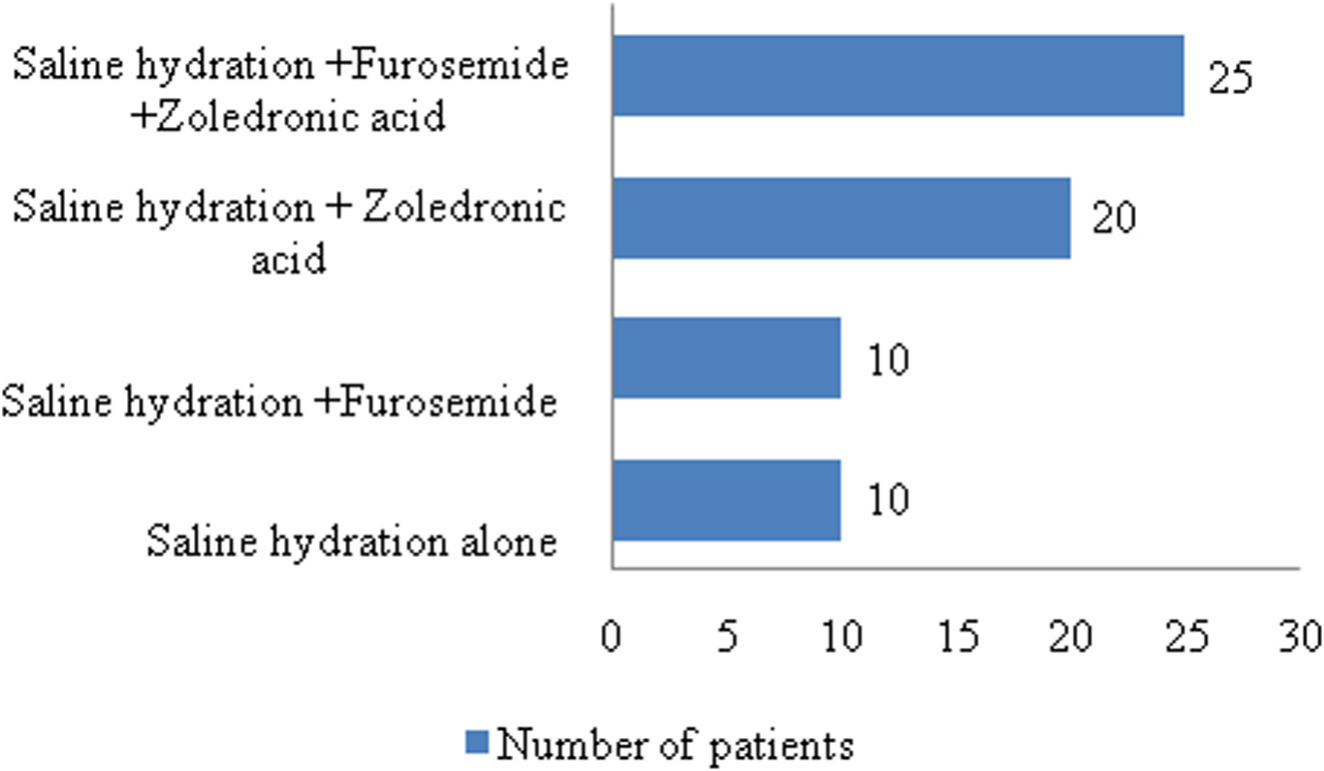
Description of the treatment protocols in the study population.

The topographic evaluation concluded to a parathyroid nodule in 61 patients and parathyroid hyperplasia in four patients. Surgical treatment was indicated in all patients. A preoperative medical antihypercalcaemic therapy was prescribed to all patients. Treatment protocols are described in figure 1.

Two patients underwent haemodialysis at the emergency room before being transferred to our department. The indication of haemodialysis was life‐threatening hypercalcaemia with cardiac arrhythmia in the first patient and neurological complications in the second patient.

Medical treatment modalities and their results are shown in Table 2. Intravenous normal saline hydration was prescribed alone in all patients with an infusion of two to four litres per day for 3.1 ± 1.7 days. Forty‐four patients received 3–4 L/24 h and 21 patients received 2 L/24 h. Serum calcium levels decreased in all patients after hydration but did not reach normal levels. The mean Δ calcium was of 0.27 ± 0.14 mmol/L (extremes: 0.025–0.62 mmol/L). There was no significant difference between patients who received 2 L/24 h and those who received 3–4 L/24 h.

**TABLE 2:** Modalities and results of medical treatment of severe hypercalcaemia.

	*n*	Dose	Duration (days)	Calcaemia level (mmol/L)			Urea level (mmol/L)			Creatinine level (μmol/L)		
				Before	After	*p*	Before	After	*p*	Before	After	*p*
IV Saline hydration (L/day)	65	4 ± 0.52	3.1 ± 1.7	3.25 ± 0.21	2.98 ± 0.2	**<10** ^**−3**^	5.99 ± 3.66	4.66 ± 2.33	**<10** ^**−3**^	79.6 ± 28.3	70.7 ± 23	**<10** ^**−3**^
IV‐Furosemide (mg/day)	35	54.3 ± 20.3	3.4 ± 2	3.16 ± 0.23	3.25 ± 0.27	**.01**	5.49 ± 2.99	7.15 ± 3.49	**<10** ^**−3**^	74.2 ± 29.2	81.3 ± 31	**<10** ^**−3**^
IV‐Zoledronic acid (mg)												
+ saline hydration	20	4		3.2 ± 0.24	2.62 ± 0.24	**<10** ^**−3**^	5.83 ± 2.83	3.66 ± 1.33	.05	79.8 ± 33.3	72.4 ± 29.8	**.001**
+ saline hydration + furosemide	25	4		3.3 ± 0.27	2.74 ± 0.19	**<10** ^**−3**^	6.49 ± 2.99	4.66 ± 2.16	**.001**	75.3 ± 26.2	68.6 ± 21.1	**.021**
Total	45	4		3.25 ± 0.26	2.68 ± 0.22	**<10** ^**−3**^	6.33 ± 2.83	4.33 ± 1.99	**<10** ^**−3**^	76.5 ± 28.1	69.5 ± 23.7	**.001**

Abbreviation: *n*, number of patients.

The difference is statistically significant in bold.

In addition to saline hydration, furosemide was prescribed in 35 patients at a mean dose of 54.3 ± 20.3 mg/day (extremes: 40–120 mg/day) during 3.4 ± 2 days. It resulted in an increase in serum calcium level in 63% of cases, a decrease in 34%, and no change in 3% of cases. The mean Δ calcium was +0.09 ± 0.21 mmol/L. The increase in serum calcium level was positively correlated with the increase in serum creatinine level after furosemide administration (*r* = 0.462, *p* = 0.004). There was no correlation between the increase in serum calcium level and the dose of furosemide (*r* = −0.041; *p* = 0.815).

Forty‐five patients received intravenous zoledronic acid (4 mg). Serum calcium levels decreased in all patients and reached normal rates in 27 of them (60%). The mean Δ calcium was of 0.57 ± 0.27 mmol/L (extremes: 0.07–1.22). The mean duration required to reach the lowest level of calcium after bisphosphonates was 6 ± 3.9 days (extremes: 3–22 days).

**FIGURE 2:**
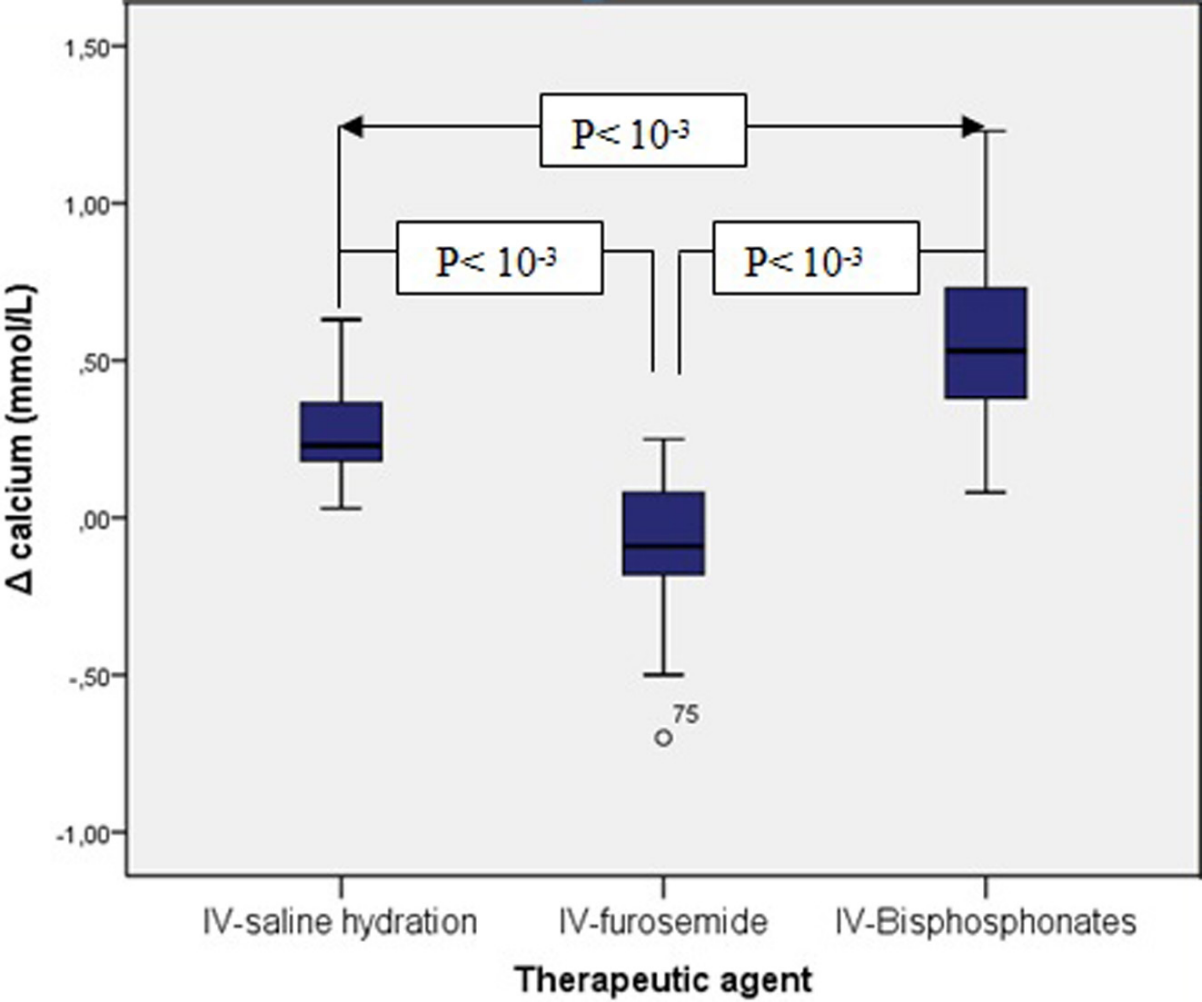
Variation in calcium levels after hydration, furosemide, and zoledronic acid.

**FIGURE 3:**
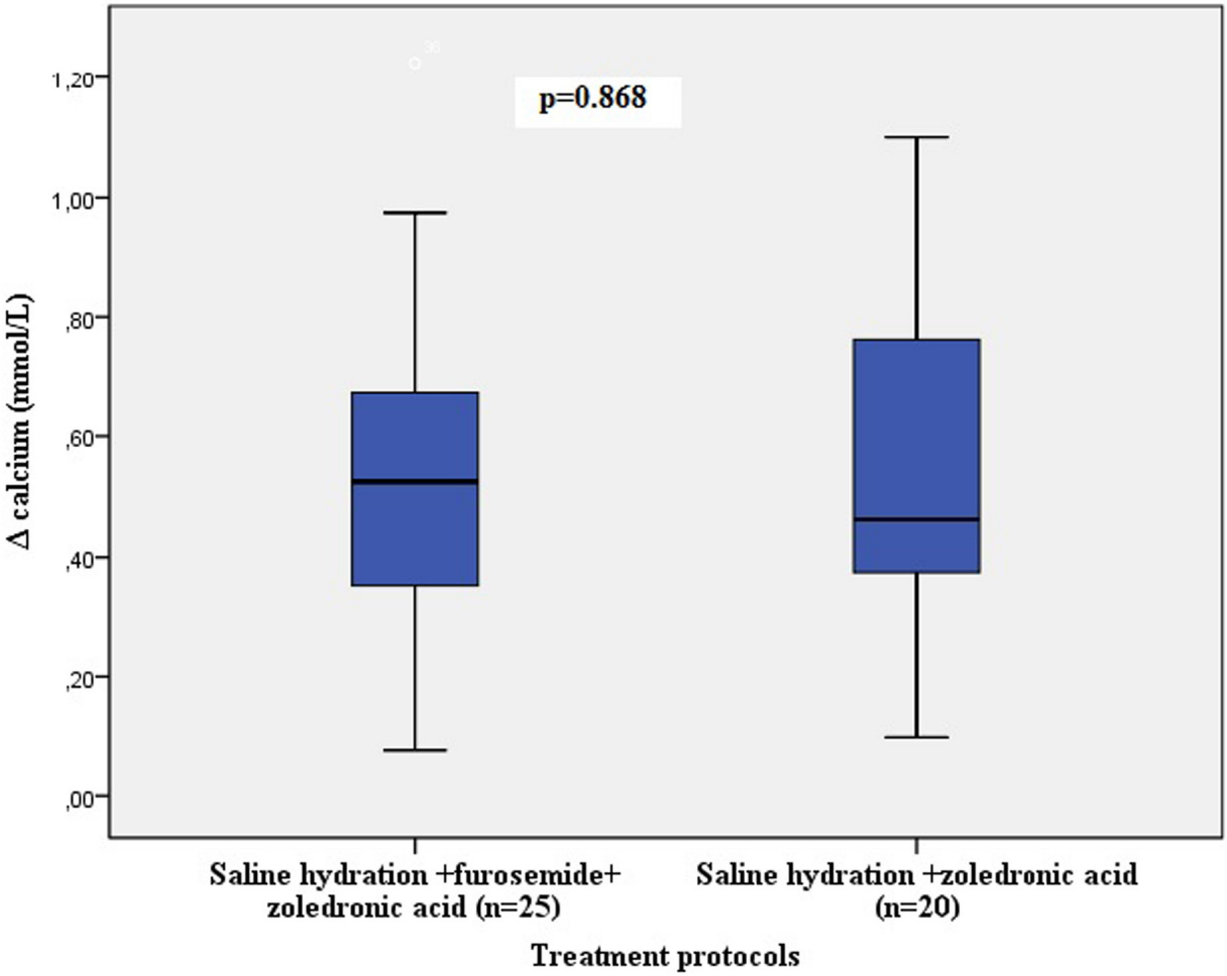
Changes in serum calcium levels in patients receiving zoledronic acid and saline hydration with or without furosemide.

**FIGURE 4:**
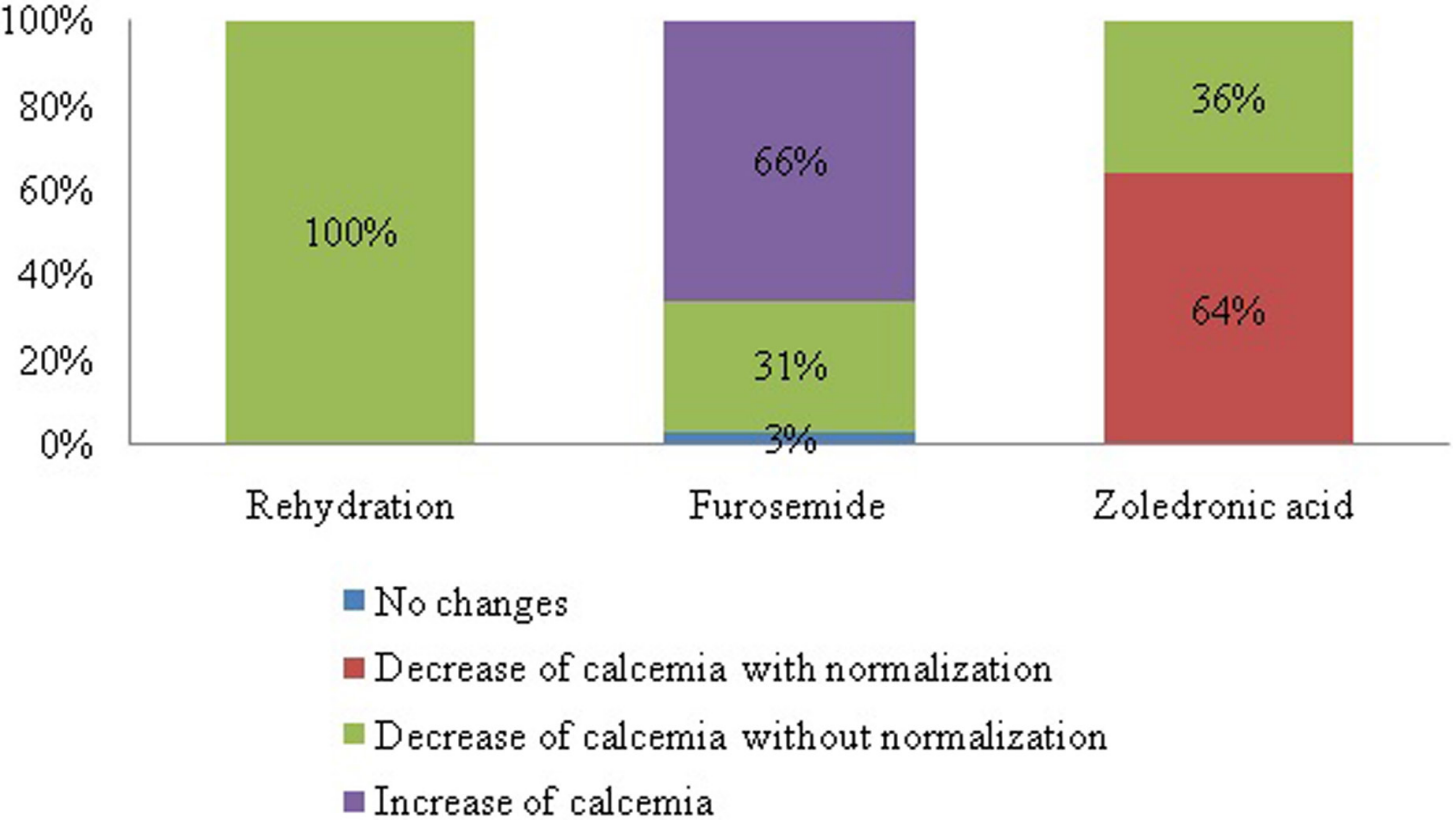
Percentage of patients with normal, decreased, or increased calcium levels after each therapeutic agent.

Figure 2 represents the variations in serum calcium levels after hydration, furosemide, and zoledronic acid administration. Figure 3 shows the changes in serum calcium levels in patients receiving zoledronic acid and saline hydration with or without furosemide. Figure 4 shows the percentage of patients with normal, decreased, or increased calcium levels after each therapeutic agent.

## 4 DISCUSSION

Our findings demonstrated a significant decrease in serum calcium levels with hydration and bisphosphonates. Meanwhile, a significant increase in serum calcium levels was observed after the use of furosemide and was significantly and positively correlated with the increase in serum creatinine level.

Hypercalcaemia may result from three possible mechanisms: increased renal tubular calcium reabsorption, stimulation of osteoclast‐mediated bone resorption, and increased renal synthesis of 1.25 (OH)2D3, which in turn promotes enhanced intestinal calcium absorption.^4^ Primary hyperparathyroidism represents one of the most frequent causes of hypercalcaemia. Severe hypercalcaemia defined by a total calcaemia greater than 3.5 mmol/L (14 mg/dl) or greater than 3 mmol/L (12 mg/dl) with signs of poor tolerance, is a life‐threatening condition.

Dehydration is a constant complication of severe hypercalcaemia. It results from polyuria and digestive disorders. It may contribute to the increase in the serum calcium level by reducing calcium clearance. Therefore, the cornerstone of initial management of severe hypercalcaemia consists of restoring extracellular volume with 0.9% normal saline solution. The objective of this measure is to restore blood volume and to re‐establish normal renal function in case of acute renal failure. Several mechanisms can explain the decrease in the calcaemia level with intravenous saline load: the release of hemoconcentration, the enhancement of calcium excretion by increasing glomerular filtration, and the calciuretic effect of sodium chloride leading to the decrease in the tubular reabsorption of calcium.^5^ The rate of fluid to infuse is based on the degree of hypercalcaemia, the severity of dehydration, the patient's age, and the associated comorbidities, in particular cardiac and renal failure. An initial flow of 200 to 300 ml/h is needed and subsequently adjusted to maintain a urine output of 100 to 150 ml/h. The recommended regimen is to administer 4–6 litres of isotonic saline daily in patients without heart or renal failure.^6^ Strict monitoring is indicated during saline hydration because of the risk of fluid overload and hypokalaemia.^6^, ^7^

Saline hydration alone is rarely sufficient to reach normal calcium levels in patients with severe hypercalcaemia. It is estimated that with saline hydration, the serum calcium concentration decreases by about 0.4 to 0.6 mmol/L.^8^, ^9^ In our study, all patients had a decrease in serum calcium levels with a mean value of 0.27 ± 0.14 mmol/L (extremes: 0.025–0.62 mmol/L) after saline hydration. However, normalization of calcium levels did not occur in any patient.

Once the intravascular volume is restored to normal, loop diuretics can be used in patients with severe hypercalcaemia to obtain a further reduction of calcium levels and to prevent fluid overload.^10^ Furosemide is a potent natriuretic that increases calcium excretion by inhibiting calcium reabsorption in the thick ascending limb of the loop of Henle.^8^ Before the advent of bisphosphonates, furosemide was an attractive alternative in the management of severe hypercalcaemia.^11^, ^12^, ^13^ However, recent data do not support its routine use in the management of severe hypercalcaemia.^14^ Suki et al.,^15^ in a study including eight patients with hypercalcaemia ranging between 3.07 and 4.6 mmol/L treated with intravenous furosemide, showed normalization of serum calcium levels in only three patients. The mean decrease in serum calcium was 0.085 mmol/L, ranging from 0.057 to 0.095 mmol/L. LeGrand et al.,^13^ in a narrative review including 37 patients (39 treatment episodes) with hypercalcaemia treated with furosemide, reported calcium level normalization in 14 of 39 episodes. This normalization occurred quickly in 6 and 12 h only in two cases. The average of furosemide dosages used was 1120 mg over 24 h, with a dose range of 240–2400 mg, and the duration of the therapy varied between 6 h and 12 days. Lower furosemide doses (40–60 mg/day, orally) did not achieve normalization at 12 days.^15^ Based on these findings, LeGrand et al.^13^ concluded that furosemide should be relegated to the management of fluid overload. On the contrary, Robey et al.^16^ considered that furosemide remains an important tool in the management of severe hypercalcaemia. Its efficiency depends on adequate attention to volume status and fluid balance before and during furosemide administration.^16^

In our study, calcaemia did not reach normal ranges in any patient after furosemide administration but increased in 63% of the cases. The mean increase in calcaemia was 0.09 ± 0.21 mmol/L. Furthermore, the use of diuretics can induce metabolic complications such as hypokalaemia, hypophosphataemia, hypomagnesaemia, hypernatraemia, and metabolic acidosis.^13^ Thus, appropriate monitoring and supplementation of these disorders are necessary.

In addition to the general measures, therapeutic agents that act on the main pathophysiological mechanism of hypercalcaemia which is the increased bone resorption should be prescribed in patients with persistent severe hypercalcaemia.^6^, ^7^ Bisphosphonates are natural analogs of pyrophosphates that bind to hydroxyapatite and act on osteoclasts by inhibiting their function and reducing their viability.^7^, ^17^ Oral bisphosphonates are poorly absorbed, with 1% or less of the administered dose being taken up.^18^ Thus, intravenous bisphosphonates are recommended in the management of severe hypercalcaemia.^7^, ^19^ The use of bisphosphonates in the treatment of malignant hypercalcaemia is well established. Intravenous infusion of pamidronate normalizes serum calcium for several days to weeks in 70%–100% of patients, although its onset of action is delayed for 1 ± 2 days.^18^ However, few published studies dealt with the use of bisphosphonates in hyperparathyroid crises. Han et al.,^20^ in a study including 14 patients with primary hyperparathyroidism complicated by hypercalcaemia crisis, reported that serum total calcium levels decreased from 3.85 ± 0.50 mmol/L to 2.86 ± 0.39 mmol/L in 1.4 ± 0.6 days after intravenous bisphosphonates. Serum calcium levels were kept below 3.50 mmol/L for 10.1 ± 8.5 days. Authors showed the absence of a significant difference in the magnitude of decrease in serum calcium levels among the patients treated either with pamidronate, ibandronate, or zoledronic acid.^20^ In the study by Phitayakorn et al.,^1^ including eight patients with hyperparathyroid crisis, six patients were treated with 60–90 mg of intravenous pamidronate, and one patient was treated with 4 mg of zoledronic acid. Isotonic sodium chloride and furosemide, in combination with intravenous bisphosphonates, resulted in a calcium decrease from 4.05 ± 0.40 to 2.95 ± 0.40 mmol/L.^1^

In our study, calcium levels decreased in all patients and reached normal ranges in 60% of them. The mean decrease in serum calcium was 0.57 ± 2.27 mmol/L [extremes: 0.075–1.22]. Because bisphosphonates require an average of 48 h to take effect, the routine approach is to initiate treatment with saline hydration while bisphosphonates act.^13^ Nevertheless, the use of bisphosphonates can induce side effects like flu‐like symptoms, fever, headache, myalgia, and even osteonecrosis of the jaw.

Other drugs such as calcimimetics and denosumab were also reported to reduce serum calcium levels in patients with primary hyperparathyroidism before surgical treatment. Calcimimetics represent a class of drugs that stimulate the activity of the extracellular calcium receptor resulting in a reduction of both PTH and calcium levels. In a study including 23 patients with primary hyperparathyroidism with hypercalcaemia >12.5 mg/dl (3.125 mmol/L), Misiorowski et al.^21^ demonstrated the efficacy of cinacalcet in reducing serum calcium levels in 19 patients (83%) with a normocalcaemia in 55% of patients. Calcimimetics were not used in our patients because they are not available in our country.

Denosumab is a receptor activator of nuclear factor kappa‐Β ligand inhibitor that decreases bone resorption. In a study including 10 patients with severe hypercalcaemia secondary to a primary hyperparathyroidism, Eremkina et al.^22^ showed that denosumab is a useful tool to reduce calcium level before surgery or if surgery is contraindicated.

Although our study has several limitations, such as its retrospective design and the relatively small number of included patients, it provided a detailed assessment of the impact of hydration, furosemide, and bisphosphonates in the management of severe hypercalcaemia secondary to primary hyperparathyroidism.

## 5 CONCLUSION

Severe hypercalcaemia is an endocrine emergency that can have severe neurological, cardiac, and renal consequences. Thus, appropriate and prompt management is necessary. Our results showed the absence of a significant additional effect of furosemide on serum calcium levels in patients with severe hypercalcaemia secondary to primary hyperparathyroidism when compared with the effect of saline hydration alone. However, zoledronic acid was more potent. Thus, appropriate normal saline hydration with immediate intravenous bisphosphonates infusion should be considered in the management of severe hypercalcaemia in patients with primary hyperparathyroidism.

## AUTHOR CONTRIBUTIONS

**Ibtissem Oueslati:** Conceptualization (lead); data curation (equal); formal analysis (equal); investigation (equal); methodology (equal); project administration (equal); resources (equal); software (equal); supervision (equal); validation (equal); visualization (equal); writing – original draft (equal). **Asma Kardi:** Data curation (equal); formal analysis (equal); investigation (equal); methodology (equal); software (equal); writing – original draft (equal). **Meriem Yazidi:** Data curation (supporting); formal analysis (supporting); investigation (supporting); methodology (supporting); supervision (supporting); validation (supporting). **Sahar Abidi:** Data curation (supporting); formal analysis (supporting); investigation (supporting); methodology (supporting); validation (supporting). **Fatma Chaker:** Data curation (supporting); formal analysis (supporting); investigation (supporting); methodology (supporting); validation (supporting). **Seifeddine Mellassi:** Data curation (supporting); formal analysis (supporting); investigation (supporting); methodology (supporting); validation (supporting). **Melika Chihaoui:** Conceptualization (supporting); data curation (supporting); formal analysis (supporting); investigation (supporting); methodology (supporting); resources (supporting); supervision (equal); validation (equal); visualization (equal); writing – review and editing (equal).

## FUNDING INFORMATION

This research did not receive any specific grant from funding agencies in the public, commercial, or not‐for‐profit sectors.

## CONFLICT OF INTEREST

The authors declare that they have no conflict of interest.

## Data Availability

The data used to support the findings of this study are available from the corresponding author upon reasonable request.

## References

[1] Phitayakorn R , McHenry CR . Hyperparathyroid crisis: use of bisphosphonates as a bridge to parathyroidectomy. J Am Coll Surg. 2008;206(6 suppl):S1106‐S1115. 10.1016/j.jamcollsurg.2007.11.010 18501807

[2] Beck W , Lew JI , Solórzano CC . Hypercalcemic crisis in the era of targeted parathyroidectomy. J Surg Res. 2011;171:404‐408. 21658721 10.1016/j.jss.2011.04.010

[3] Cannon J , Lew JI , Solórzano CC . Parathyroidectomy for hypercalcemic crisis: 40 years experience and long‐term outcomes. Surgery. 2010;148(4 suppl):S807‐S812. 10.1016/j.surg.2010.07.041 20800863

[4] Dandurand K , Ali DS , Khan AA . Primary hyperparathyroidism: a narrative review of diagnosis and medical management. J Clin Med. 2021;10(8 suppl):S1604. 10.3390/jcm10081604 PMC8068862 33918966

[5] Rossi E , Perazzoli F , Negro A , et al. Acute effects of intravenous sodium chloride load on calcium metabolism and on parathyroid function in patients with primary aldosteronism compared with subjects with essential hypertension. Am J Hypertens. 1998;11:8‐13. 9504444 10.1016/s0895-7061(97)00366-x

[6] Walsh J , Gittoes N , Selby P . Society for Endocrinology clinical committee. Society for Endocrinology endocrine emergency guidance: emergency management of acute hypercalcaemia in adult patients. Endocr. Connect. 2016;5(5 Suppl):G9‐G11. 10.1530/EC-16-0055 PMC5314807 27935816

[7] Bilezikian JP . Management of acute hypercalcemia. N Engl J Med. 1992;326:1196‐1203. 1532633 10.1056/NEJM199204303261806

[8] Guitton C , Renard B , Gabillet L , Villers D . Dyscalcémie aux urgences. Réanimation. 2002;11:493‐501.

[9] Oueslati I , Rached A , Chihaoui M , et al. Prise en charge de l'hypercalcémie sévère. Méd Thér. 2018;24(2 Suppl):S89‐S95.

[10] Baguet JC , Rampon S , Bussière JL , et al. Treatment of acute hypercalcemia with furosemide. Rev Rhum mal Osteoartic. 1972;39(8 Suppl):S531‐S535. 5084224

[11] Helzberg J , Pinnick R , Grantham JJ . Refractory hypercalcemia. Management with intravenous fluids and furosemide guided by swan‐Ganz monitoring. J Kans Med Soc. 1983;84:16‐17. 40. 6827171

[12] Fillastre JP , Humbert G , Leroy J . Treatment of acute hypercalcemia with furosemide. Curr Ther Res Clin Exp. 1973;15:641‐649. 4201547

[13] LeGrand SB , Leskuski D , Zama I . Narrative review: furosemide for hypercalcemia: an unproven yet common practice. Ann Intern Med. 2008;149:259‐263. 18711156 10.7326/0003-4819-149-4-200808190-00007

[14] Suki WN , Yium JJ , Von Minden M , Saller‐Hebert C , Eknoyan G , Martinez‐Maldonado M . Acute treatment of hypercalcemia with furosemide. N Engl J Med. 1970;283(16 Suppl):S836‐S840. 10.1056/NEJM197010152831603 5458033

[15] Caron C . [Treatment of hypercalcemia using furosemide]. *Union Med Can*. 1975;104:272–275. 1162763

[16] Robey RB , Lash JP , Arruda JA . Does furosemide have a role in the management of hypercalcemia? Ann Intern Med. 2009;150(2 Suppl):S146‐S147. 10.7326/0003-4819-150-2-200901200-00020 19153420

[17] Russell RG , Rogers MJ . Bisphosphonates: from the laboratory to the clinic and back again. Bone. 1999;25:97‐106. 10423031 10.1016/s8756-3282(99)00116-7

[18] Vasikaran SD . Bisphosphonates: an overview with special reference to alendronate. Ann Clin Biochem. 2001;38(Pt 6):608‐623. 11732644 10.1258/0004563011901037

[19] Ahmad S , Kuraganti G , Steenkamp D . Hypercalcemic crisis: aclinical review. Am J Med. 2015;128:239‐245. 25447624 10.1016/j.amjmed.2014.09.030

[20] Han GY , Wang O , Xing XP , et al. [The efficacy and safety of intravenous bisphosphonates in the treatment of primary hyperparathyroidism complicated by hypercalcemia crisis]. *Zhonghua Nei Ke Za Zhi*. 2009;48(9):729–733. 20079207

[21] Misiorowski W , Zgliczyński W . Cinacalcet as symptomatic treatment of hypercalcaemia in primary hyperparathyroidism prior to surgery. Endokrynol Pol. 2017;68(3):306‐310. 28660989 10.5603/EP.2017.0023

[22] Eremkina A , Krupinova J , Dobreva E , et al. Denosumab for management of severe hypercalcemia in primary hyperparathyroidism. Endocr Connect. 2020;9(10):1019‐1027. 33112830 10.1530/EC-20-0380 PMC7707828

